# Synchronous Endometrial and Ovarian Adenocarcinomas in a 43-Year-Old Patient Following Infertility Treatment: A Case Report

**DOI:** 10.3390/diagnostics15060670

**Published:** 2025-03-10

**Authors:** Małgorzata Gajewska, Barbara Suchońska, Joanna Blok, Wanda Gajzlerska-Majewska, Artur Ludwin

**Affiliations:** 1st Department and Clinic of Obstetrics and Gynecology, Medical University of Warsaw, 02-091 Warsaw, Poland; malgorzata.gajewska@wum.edu.pl (M.G.); joanna.blok@wum.edu.pl (J.B.); wanda.gajzlerska-majewska@wum.edu.pl (W.G.-M.); artur.ludwin@wum.edu.pl (A.L.)

**Keywords:** synchronous cancer, ovarian cancer, endometrial cancer, infertility, assisted reproductive technology, synchronous adenocarcinomas, ovarian adenocarcinoma, endometrial hyperplasia, conservative treatment, LNG-IUS

## Abstract

**Background and Clinical Significance:** This study presents a case of a 43-year-old female with a long history of infertility, treated for uterine leiomyoma and endometrial hyperplasia, over a total observation period of 42 months. **Case Presentation:** Levonorgestrel intrauterine device (LNG-IUD) therapy, as a first and subsequent line of treatment, was introduced. The patient also received medroxyprogesterone acetate oral treatment. Finally, she underwent surgery for an ovarian tumor that appeared to be an ovarian adenocarcinoma concurrent with endometrial cancer. After the removal of the reproductive organ, the patient was diagnosed with synchronous low-grade endometrioid adenocarcinoma in the endometrium and a concurrent grade 2 (G2) endometrioid adenocarcinoma in the left ovary. **Conclusions:** The prognosis and further management largely depend on whether these are two individual neoplasms or one metastatic tumor. Considering the young age of the patients, an early disease stage, a low grade of both cancers, and favorable prognosis, most synchronous endometrial and ovarian cancers are identified as two independent primary tumors. The diagnosis of a multi-focal neoplasm is important, as in patients with endometrial cancer and ovarian metastasis, the 5-year survival rate is 30–40%, whereas in the case of individual neoplasms, it is 75–80%.

## 1. Introduction

Concurrent endometrial and ovarian adenocarcinomas are found in 5% of women with endometrial cancer and in 10% of patients with ovarian cancer [1]. Synchronous ovarian and endometrial cancers, although rare, account for 50% to 70% of synchronous neoplasms of the reproductive organ [2]. Histopathological examination in both cases typically presents the endometrioid type [3]. Table 1 presents publications, including multicenter and single-center retrospective reviews, as well as case reports related to the issue. Research reports indicate that more than 50% of these patients suffer from infertility [4,5,6,7,8,9,10,11,12,13]. The prognosis and further management largely depend on whether these are two individual neoplasms or one metastatic tumor. Considering the young age of patients, early disease stage, low grade of both cancers, and favorable prognosis, most synchronous endometrial and ovarian cancers are identified as two independent primary tumors [14,15]. At present, the risk factors for the development of gynecological neoplasms are well established. One of them is infertility, recognized by the World Health Organization as a social disease. Numerous authors suggest that infertility, as an individual disease, may contribute to the development of endometrial, ovarian, and breast cancers [16,17,18,19,20]. Polycystic ovary syndrome (PCOS), anovulatory cycles, obesity, and insulin resistance not only impede conception but also serve as risk factors for the development of endometrial cancer. Multiple ovarian punctures, along with extremely high levels of sex hormones and gonadotropins associated with in vitro fertilization, may induce carcinogenesis in gonads [21,22,23]. The fact that the majority of women treated for infertility and diagnosed with a malignant neoplasm of the reproductive organ are young, usually under 45 years of age, creates a significant problem. Approximately 5% of patients diagnosed with endometrial cancer are under 40 years old, while 10% are under 45. Most of them have a history of infertility, obesity, PCOS, and anovulatory cycles [24]. Synchronous cancers occur rarely; however, awareness and the ability to differentiate them from metastatic tumors are crucial for patient counseling. A comprehensive understanding of such conditions should be prevalent, and further research is required to elucidate every detail of the disease.

## 2. Case Report

A 43-year-old nulliparous female, body mass index (BMI) 21.9, was admitted to the 1st Department of Obstetrics and Gynecology at the Medical University of Warsaw for surgical treatment of ovarian cancer. Regular menstrual cycles of 25 days with light bleeding were observed. No other coexisting medical conditions were identified. The patient presented with a long history of infertility treatment and the enucleation of four uterine leiomyomas at 35 years of age, followed by two unsuccessful in vitro fertilizations. In the past, at the age of 38, the patient underwent diagnostic laparoscopy and uterine curettage. A microscopic examination revealed atypical endometrial hyperplasia. The applied treatment method was Levonorgestrel-releasing intrauterine system (LNG-IUS) for six months. A follow-up verification of the endometrium demonstrated fragments of endometrium with hyperplasia without clear signs of atypia. The patient declined to continue the treatment. After 18 months, the patient returned to the Infertility Clinic. Due to a suspicion of an endometrial polyp, a hysteroscopy was performed. A microscopic evaluation revealed endometrial hyperplasia without atypia. The patient was prescribed 10 mg of oral medroxyprogesterone acetate a day for three months. A histopathological examination of the endometrium after treatment demonstrated that endometrial hyperplasia persisted. Surgical treatment was suggested to the patient. Since the patient had no children, no atypical cells were found in the microscopic evaluation and following the patient’s request, another therapy with Levonorgestrel-releasing intrauterine system was conducted for six months. After that period, the LNG-IUS was removed, and a Pipelle endometrial biopsy did not reveal hyperplasia. Three months later, the patient underwent two in vitro fertilization procedures using frozen embryos; however, neither was successful. After six months, the patient returned for a follow-up appointment. A gynecological examination revealed an abnormal mass in the left adnexa, interpreted in the ultrasound examination as a solid-cystic tumor of the left ovary. The patient was referred to the Department of Surgical Gynecology.

**Figure 1:**
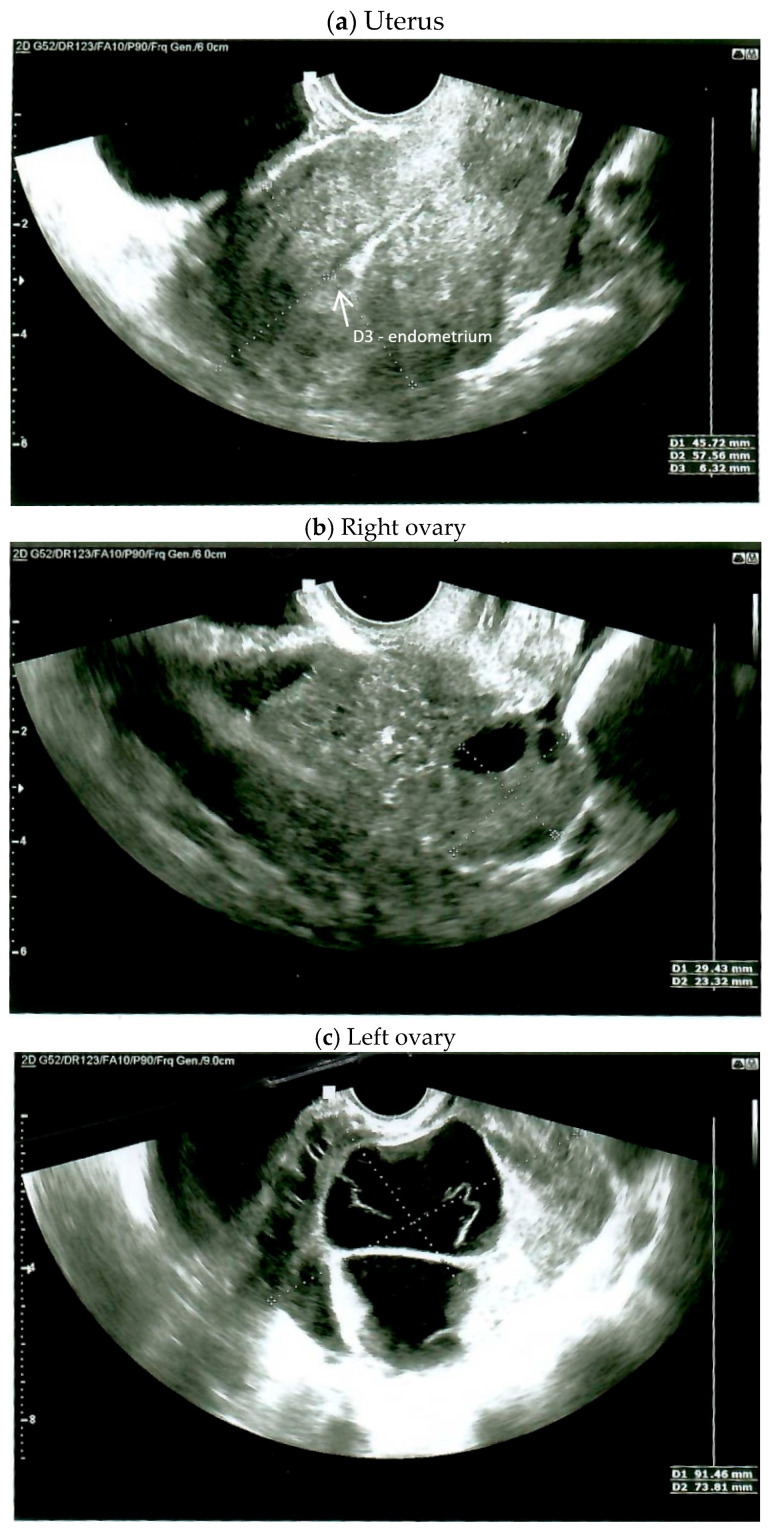
Ultrasound on the day of admission.

On the day of admission, the female was in good general condition. A gynecological examination demonstrated a normal-sized, retroverted body of the uterus, displaced to the right, with limited mobility. A tumor approximately 10 cm in diameter, with limited mobility, was detected behind the uterus on the left side. The ultrasound findings were as follows: the body of the uterus was homogeneous, measuring 45 × 57 mm; the endometrium was 6.3 mm thick and a small amount of free fluid behind the uterus was detected (Figure 1a); the right ovary measured 29 × 23 mm (Figure 1b); and the left ovary appeared as a solid-cystic tumor of 91 × 73 mm (Figure 1c). A multiple vascular pattern in the Doppler ultrasound was confirmed. The blood tests revealed a cancer antigen 125 (CA 125) level of 437 mIU/mL, a human epididymis protein 4 (HE-4) level of 481 pmol/L, and a Risk of Ovarian Malignancy Algorithm (ROMA) score of 95.6%. The patient qualified for surgical treatment.

After the abdominal wall was opened, a small amount of serous fluid was found and collected for testing. The body of the uterus was of normal size, with a smooth surface and limited mobility. The adnexa on the right appeared normal in gross appearance. The left ovary presented as a solid-cystic tumor of approximately 10 cm in diameter, adhering to the posterior lamina of the broad uterine ligament and the sigmoid loop. The fallopian tube was stretched over the tumor. Both lobes of the liver were smooth, and the parietal peritoneum and intestines showed no lesions in gross appearance. No resistance was found in the periaortic area. The omentum was unremarkable. Adhesions were removed. During preparation, the tumor capsule burst, spilling bloody content. The left adnexa were removed and sent for intraoperative testing, which confirmed ovarian cancer. The uterus, right adnexa, regional lymph nodes, and omentum were removed. Peritoneal tissue samples were collected from the paracolic gutters on both the right and left sides. The surgery and the post-operative period were uncomplicated. The patient was discharged in generally good condition on the third day following the procedure.

**Figure 2:**
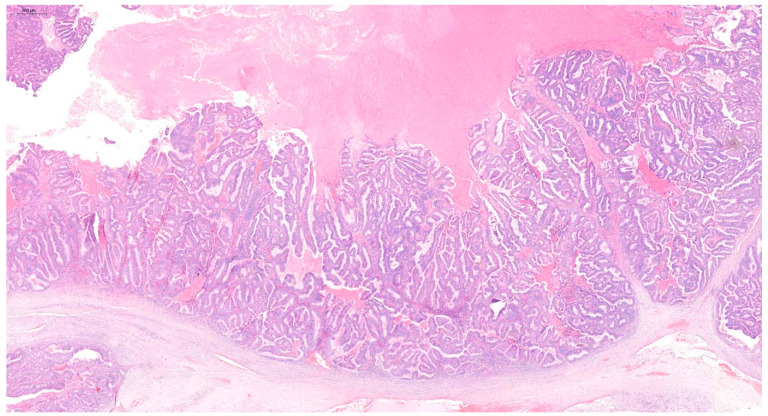
Hematoxylin and eosin (H&E) staining of the G2 endometrioid cancer of the ovary.

**Figure 3:**
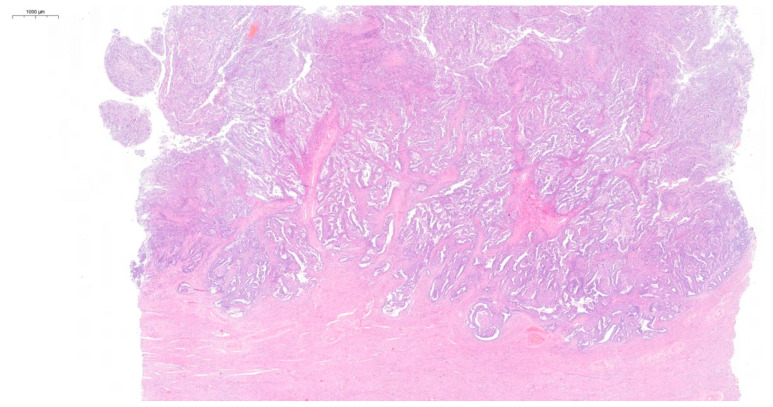
Hematoxylin and eosin (H&E) staining of the complex endometrial hyperplasia with atypia and low-grade endometrioid cancer.

Postoperative histopathological examination revealed grade 2 (G2) endometrioid cancer of the left ovary (Figure 2). No signs of angioinvasion or invasion of the nerve trunks were found. The cancer structure penetrated the surface of the ovarian capsule. The cancer cell immunophenotype was cytokeratin 7 (CK7) (+), cytokeratin 20 (CK20) (−), estrogen receptor (ER) (+), Wilms’ tumor gene 1 (WT1) (−). The left fallopian tube was normal. Functional cysts and a corpus luteum cyst were found in the right ovary. The right fallopian tube was normal. In the body of the uterus, complex endometrial hyperplasia with atypia was observed, as well as small foci of low-grade endometrioid cancer (Figure 3). The cancer was limited to the endometrium. No signs of angioinvasion or invasion of the nerve trunks were found. Leiomyomas and adenomyosis were discovered in the body of the uterus. In the left parametrium, a microfocus of cancer structure was present. No cancer was found in the right parametrium. No metastasis was observed in the lymph nodes. No cancer was found in the omentum or in the peritoneal tissue samples. Neoplastic cells were observed in the peritoneal fluid. The histopathological findings indicated two individual cancer foci—endometrial and ovarian. The stages of the tumors were endometrial adenocarcinoma IA and ovarian adenocarcinoma IIB. The patient qualified for chemotherapy. The patient’s condition was under control for five years following the surgery. During the first two years, a follow-up appointment took place every three months, then once every six months. The patient has been free of recurrence until now.

## 3. Discussion

This case report outlines the course of synchronous endometrial and ovarian cancer in a 43-year-old woman following unsuccessful infertility treatment. After the removal of the reproductive organ, the patient was diagnosed with synchronous low-grade endometrioid adenocarcinoma in the endometrium, along with a concurrent G2 endometrioid adenocarcinoma in the left ovary. The diagnosis of a multifocal neoplasm is important, as patients with endometrial cancer and ovarian metastasis exhibit a 5-year survival rate ranging from 30% to 40%. Conversely, when considering individual neoplasms, the 5-year survival rate is notably higher, averaging between 75% and 80%. On the other hand, Heitz et al. [25] postulate that the prognosis for patients with synchronous tumors is similar to that of individual diagnoses of endometrial and ovarian cancer, taking into consideration factors such as age, stage, and grade. It is worth noting that the treatment of synchronous cancers differs significantly from that of endometrial cancer with ovarian metastasis. The study conducted by Nguyen focused on the role of the Doppler ultrasound. The researchers aimed to determine the significance of combined Doppler and B-mode ultrasonography in diagnosing benign and malignant uterine intracavitary pathologies. The study found Doppler ultrasound to be more accurate than B-mode alone and proved that using both can help differentiate between the lesions. The authors emphasize the importance of Doppler flow mapping in evaluating uterine malignancy. In their research, most of the malignancies showed a Doppler ultrasound multivascular pattern [26]. Therefore, the presence of the Doppler signal in endometrial lesions is strongly related to the risk of malignancy. In the presented case, the Doppler signal was detected in the endometrial lesion.

Moreover, the researchers in 2022 evaluated the role of Doppler indices of the uterine artery as markers for endometrial cancer. In their study, the resistance index, pulsatility index, and peak systolic velocity were significantly lower in the malignant group compared to the benign group. Furthermore, these values were found to decrease with more advanced stages of endometrial cancer [27].

In this study, after the diagnosis of atypical endometrial hyperplasia, LNG-IUD therapy was introduced for six months. First-line treatment with intrauterine devices follows the RCOG and BSGE guidelines [28]. The use of the LNG-IUD in patients with atypical hyperplasia resulted in significantly higher regression rates in comparison to oral gestagen therapy [29,30,31,32,33,34]. The LNG-IUD has a direct impact on progesterone receptors in the endometrium, leading to a markedly elevated concentration of progesterone in the mucosa [35]. According to a study conducted by Gallos et al., the regression rates for IUD and oral gestagens were reported as 90% and 69%, respectively [29]. Similarly, in another study, the rate of relapses was lower following the use of LNG-IUD compared to oral gestagens (6.5% vs. 20%) [36]. The endometrial biopsy performed 18 months later demonstrated that the hyperplasia without atypia persisted. No regression of endometrial hyperplasia after 12 months of treatment is an indication for hysterectomy [28]. However, the patient did not receive treatment for 18 months, and no follow-up biopsies were performed. Therefore, it cannot be considered a complete treatment, and even less so a treatment failure or a recurrence. Recurrence of hyperplasia following therapy is associated with a higher risk of cancer, and it is another indication for surgical treatment. However, such decisions are particularly difficult in the case of nonparous women. In their study, Yu et al. repeated gestagen therapy in two patients with recurrent atypical hyperplasia, which resulted in a remission of lesions [37]. In the presented case, one could not speak of a 12-month treatment and its failure, so gestagen therapy was applied again. Due to multiple curettage procedures in the nonparous patient, an aspiration biopsy was performed, which did not reveal any lesions. In light of the conclusive findings from the histopathological examination of the endometrium, the negative result of the endometrial aspiration biopsy conducted subsequent to the removal of the intrauterine device (IUD) should be regarded with skepticism. It should be emphasized that the Pipelle biopsy collects material from only 4% of the endometrial surface [38]. The created negative pressure allows for the collection of the majority of pathological tissue, characterized by a weaker connection to the healthy tissue. However, no ideal tool for diagnosing endometrial cancer is available. A basic test, such as fractional curettage of the uterus, provides an assessment of more than half of the uterine cavity in less than 60% of women [39]. Moreover, curettage, considered a minor procedure, is frequently performed by younger and less experienced physicians. All of these factors may contribute to a certain percentage of false-negative results. The limitations of diagnostic methods should alert us, and awareness of their flaws should lead to curettage procedures being performed carefully by trained medical professionals. This may increase the sensitivity of the method, especially in the presence of single focal lesions. The presented patient underwent multiple procedures involving assisted reproduction techniques, which, according to numerous authors, may contribute to the development of neoplasms in the reproductive organ [21,22,23]. However, the findings from many studies investigating this problem are inconsistent. Numerous authors report an increased risk of ovarian cancer in women receiving infertility treatment compared to the general population. It is of great importance, as no such correlation can be observed for the population of infertile women who do not receive treatment [40,41,42,43]. It is crucial to emphasize that fertility-preserving treatment in endometrial hyperplasia with atypia and early-stage endometrial cancer represents a significant concern, as 4% of women affected are of reproductive age. About 70% of women in this group are nulliparous at the time of diagnosis. Therefore, making a decision regarding fertility preservation is a major challenge. This is not only a medical dilemma, but also an ethical and social one. On the one hand, we give a chance for motherhood, and on the other hand, we risk cancer progression [44]. In general, complex atypical hyperplasia has higher response rates, lower recurrence rates, and a lower risk of persistent disease compared to endometrial cancer when treated with progestin [45]. One of the main factors considered for fertility preservation treatment is a high tumor grade (G1). Endometrial cancer grade G2 is strongly linked to worse obstetric outcomes compared to grade G1 [46]. A group of researchers led by Giampaolino presents the conservative management of 84 patients diagnosed with IA G2 stage endometrial cancer, achieving complete remission in 64% women—a total of 22 patients became pregnant, resulting in eight deliveries at term. The oncologic outcomes showed that patients who became pregnant experienced recurrence later than those who did not conceive [36]. There remains considerable controversy regarding the synchronous occurrence of endometrial and ovarian cancers, and specifically whether they represent two distinct conditions or a single disseminated cancer. Taking into account the early stages of progression in most patients, high grade, and favorable course, many authors consider them to be two independent neoplasms. A preoperative ultrasound examination typically does not facilitate the identification of a common or independent origin of tumors. Nevertheless, Moro et al. believe that in cases of synchronous tumors, the ovarian tumor is usually unilateral and solid-cystic, in contrast to metastatic tumors from the endometrium to the ovary, which are more often solid and bilateral [7]. In the case of synchronous tumors, myometrial infiltration is also less common than in endometrial cancer with ovarian metastases. On the one hand, there are strictly defined histopathological criteria for the diagnosis of synchronous tumors, supporting their separate origins. Histologic dissimilarity of the tumors, superficial myometrial invasion, no vascular space invasion of endometrial and ovarian tumors, unilateral ovarian tumors, an absence of other evidence of spread of endometrial and ovarian tumors, ovarian tumor located in parenchyma, and the presence of ovarian endometriosis argue for the conclusion of two independent tumors. On the other hand, genotyping of nuclear and mitochondrial DNA in synchronous endometrial and ovarian cancers indicates a common origin [11,47,48]. A group of scientists led by Schultheis postulates that synchronous endometrial and ovarian cancers share the same origin. According to the authors, early endometrial cancer is implanted in an ovary due to the retrograde flow of cells through the fallopian tubes [1]. A study presented by Anglesio et al. describes the restricted metastasis of synchronous endometrial and ovarian cancers as clonally related. It is possible that the neoplastic cells from the primary lesion colonize exclusive, loco-regional microenvironments, without widespread dissemination. The author indicates that these tumors demonstrate very restricted metastasis, with involvement limited mainly to the endometrium and ovaries [47]. Further research is required to identify which organ has the primary lesion, as well as the direction of metastasis.

## 4. Conclusions

This case report presents a patient who underwent years of unsuccessful infertility treatment and, at the age of 43, had a reproductive organ removed due to concurrent ovarian and endometrial cancer. It is difficult to determine whether the tumors are two independent neoplasms or one misdiagnosed endometrial cancer that developed from long-treated hyperplasia of the mucosal membrane. Unfortunately, the literature suggests that infertility may contribute to hormone-dependent neoplasms. This consideration is essential when initiating frequently extended infertility treatment. During counseling, patients should be informed about the possibility of unfavorable treatment outcomes, including the development of a pathology that could lead to the loss of a reproductive organ and may pose a threat to the patient’s life.

## Figures and Tables

**Table 1:** Cases of synchronous ovarian and endometrial carcinoma.

Article	Number of Cases	Research Time	Mean Age	Endometrial Cancer Characteristics		Ovarian Cancer Characteristics	
				Histological Type	Number of Cases	Histopathological Type	Number of Cases
Song T et al. [4]	123	1995–2010	50.4	Endometrioid Non-endometrioid	100 23	Endometrioid Serous Clear-cell Mucinous Mixed Others	62 26 8 15 8 4
Sylwia DębskaSzmich et al. [5]	10	2008–2013	56	Endometrioid	10	Endometrioid Papillary cystadenocarcinoma Mucinous adenocarcinoma Undifferentiated carcinoma	7 1 1 1
Yong Kuei Lim et al. [6]	46	2000–2009	47.3	Endometrioid	46	Endometrioid Non-endometrioid	34 12
Moro F et al. [7]	51	2010–2018	57.7	Endometrioid Non-endometrioid	43 8	Endometrioid High-grade serous carcinoma	30 10
						Clear-cell carcinoma Mixed Tubal cancer Other	3 3 2 3
Wang T et al. [8]	51	2009–2017	53.96	Endometrioid	51	Endometrioid Non-endometrioid	33 18
Ma SK et al. [9]	43	2008	49	Endometrioid	43	Endometrioid or a mixed tumor with endometrioid component Non-endometrioid	30 13
Bese T et al. [10]	31	1997–2015	52.6			Endometrioid Non-endometrioid	18 13
Shin W et al. [11]	28	2006–2018	50	Endometrioid Non-endometrioid (serous clear cell)	25 3 2 1	Endometrioid Non-endometrioid Serous Clear-cell Seromucinous Mucinous Carcinosarcoma	17 11 5 2 2 1 1
Dogat A et al. [12]	1	2017	45	Endometrioid		Endometrioid	
Decavalas G, et al. [13]	1	2006	49	Endometrioid		Endometrioid	

## Data Availability

Data are available from the corresponding author upon reasonable request.
